# The role of peripheral type 2 innate lymphoid cells in bronchiolitis

**DOI:** 10.1038/s41598-021-82096-5

**Published:** 2021-01-29

**Authors:** Yong-Jun Tang, Li-Li Xie, Xiang-Rong Zheng, Chen-Tao Liu, Xia Wang

**Affiliations:** 1 Department of Pediatrics, Xiangya Hospital, Central South University, Changsha, 410008 Hunan People’s Republic of China; 2 Newborn Department of Pediatrics, Renmin Hospital of Wuhan University, Wuhan, 430000 Hubei People’s Republic of China

**Keywords:** Immunology, Chemokines

## Abstract

Our aim was to detect type 2 innate lymphoid cells (ILC2s)-related cytokines of infants with bronchiolitis by using Elisa, Liquidchip technology and RT-PCR and investigated its correlation with bronchiolitis. We recruited 26 infants with bronchiolitis and 20 healthy infants as control from Xiangya Hospital. Compared to the control group, the serum levels of interleukin-5 (IL-5) [41.99 (21.11) vs 25.70 (19.64)], IL-9 [27.04 (37.51) vs 8.30 (0.54)], IL-13 [184.05 (132.81) vs 121.75 (176.13)], IL-33 [83.70 (46.69) vs 11.23 (55.31)] and thymic stromal lymphopoietin (TSLP) [31.42 (5.41) vs 28.76 (2.56)] were significantly increased in infants with bronchiolitis (*P* < 0.05), while the level of IgE had no significant difference between the two groups [19.05 (14.15) vs 14.85 (20.2), *P* > 0.05]. The mRNA expression of IL-17RB (9.83 ± 0.35 vs 9.19 ± 0.58), TSLP (16.98 ± 2.12 vs 15.07 ± 2.25), retinoid acid receptor related orphan receptor α (7.18 ± 0.71 vs 5.46 ± 1.09) and trans-acting T-cell-specific transcription factor 3 (4.86 ± 0.66 vs 4.19 ± 0.90) were significantly increased in infants with bronchiolitis versus the control group (*P* < 0.05), while there was no statistical significance for suppression of tumorigenicity 2 (5.59 ± 0.68 vs 5.41 ± 0.87, *P* > 0.05). Our findings suggested that ILC2s possibly play a specific role in immunopathology of bronchiolitis.

## Introduction

Bronchiolitis is a common acute lower respiratory tract infection caused by different viruses. A considerable number of infants under 12 months have bronchiolitis at least once and around 2–3% of them require admission to hospital^1,2^. Wainwright C^3^ reported that about 20% of children develop bronchiolitis during the first year of life, at the same time the incidence of bronchiolitis is increasingly year by year. It is the most common cause of hospitalization of children in many countries and between 3 and 7% of them require mechanically assisted ventilation during hospitalization^4^. Therefore, bronchiolitis not only seriously harms the physical and mental health of children, but also brings a heavy burden on the family and society.

At present, the pathogenesis of bronchiolitis is still unclear and it may be related to genetics, viral infections, immunity and environmental factors. Respiratory syncytial virus (RSV) is the main pathogen of bronchiolitis in terms of viral infection, which not only directly damages airway mucosa epithelium, but also can be used as an allergen to induce immune response. Recently, Frassanito et al.^5^ investigated the T-cell response in RSV-infected pediatric patients by using a short-term whole blood intracellular staining assay, which offered a convincing approach for future clinical studies. In terms of immunology, the factors currently reported to be involved in the pathogenesis of bronchiolitis include: T helper 1/T helper 2 (Th1/Th2) imbalance^6^, Th17^7^, CD4 + CD25 + regulatory T cells^8^, CD40/CD40L^9^. The clinical manifestations and pathological changes of bronchiolitis are similar to asthma. Several studies have shown that infants and young children hospitalized for wheezing between 12 and 24 months are at high risk of developing asthma^10,11^. Yanney et al.^12^ reported that the main pathological change of bronchiolitis is small airway obstruction caused by acute inflammation. About 34–50% of children with bronchiolitis will experience secondary airway hyperresponsiveness (AHR) in the future. Type 2 cytokines that occur during the onset of asthma can also be found in bronchiolitis. All of the above indicate that bronchiolitis has a similar pathogenesis to asthma.

For a long time, it has been thought that the immunological pathogenesis of asthma is the imbalance of function and ratio of Th1/Th2 subgroup^13^, in which the function of Th1 cell is suppressed while Th2 cell is hyper-function. In 2000, Graham^14^ and others proposed for the first time that children with bronchiolitis are prone to develop asthma, which may be related to the hyper-function of Th2-derived cytokines (interleukin-4 (IL-4), IL-5, and IL-13). Since then, reports on the immunological mechanism of bronchiolitis have also shown that the expression of IL-2 and interferon-γ in serum decreased and the expression of Th2-derived cytokines (IL-4, IL-5, IL-6, IL-8, and IL-10) increased, suggesting that the pathogenesis of bronchiolitis and asthma also exist immune imbalances of Th1/Th2^6,15^.

In recent years, a new perspective on the immunological pathogenesis of asthma suggests that type 2 innate lymphoid cells (ILC2s) exist except for Th2 cells. ILC2s do not depend on T cells and secrete a large number of type 2 cytokines (such as IL-4, IL5, IL-9, and IL-13) under the function of IL-25, IL-33 and thymic stromal lymphopoietin (TSLP) to participate in asthma, virus infection and worm-host defense related with the type 2 immune response^16^. ILC2s are dependent on transcription factor inhibitor of DNA binding 2, trans-acting T-cell-specific transcription factor 3 (GATA-3), retinoid acid receptor related orphan receptorα (RORα) as well as Notch signals^17–19^, in which GATA-3 and RORα are specific transcription factors for ILC2s. Because the surface molecules of ILC2s are still not very clear, researchers mainly detect the followings to prove the presence of ILC2s: IL-25R (IL-17RB), IL-33R (T1/ST2), and TSLP expressed by ILC2s, and the transcription factors GATA-3, RORα, which are required for the maturation and differentiation of ILC2s as well as IL-5, IL-9, and IL-13 secreted by ILC2s^16–19^. In our study, we focused on detecting ILC2s-related cytokines to explore the relationship between ILC2s and the occurrence and development of bronchiolitis, to further understand the pathogenesis of bronchiolitis, and to provide a new way for the treatment of bronchiolitis.

## Methods

### Study population

The study subjects included 26 infants with bronchiolitis and 20 health children, recruited from Xiangya Hospital of Central South University. They were 2 months to 2 years old and divided into the bronchiolitis group and the control group. The clinical and demographic characteristics of the subjects with bronchiolitis and the normal controls are shown in Table 1. There was no statistical difference in gender and age between groups (*P* > 0.05, see Table 1). Parents of both patients and control subjects signed informed consent. The study was approved by the ethics committee of Xiangya Hospital and was registered in the Chinese Clinical Trial Registry (Registry ID: 20151029-08).We performed the clinical trial in accordance with the relevant guidelines and regulations.

**Table 1:** Demographic and clinical characteristics of the study subjects.

Characteristic	Bronchiolitis group (n = 26)	Control group (n = 20)	*P* value
Age (months)	10.1 (2–24)	11.3 (3–23)	> 0.05
Males	76.9%	60%	> 0.05
Days of disease	9.6 (4.7)	–	–
White blood cells (10^9^/L)	10.5 (5.6)	8.6 (1.2)	> 0.05
Neutrophils (%)	29.8 (16.5)	52.1 (6.7)	< 0.01
Lymphocytes (%)	51.4 (18.7)	35.7 (5.7)	< 0.01
Monocytes (%)	0.8 (0.48)	0.8 (0.4)	> 0.05
Eosinophils (%)	10.2 (3.3)	9.8 (2.1)	> 0.05

Data are expressed as median (range) or percentage (SEM).

The bronchiolitis group consisted of 18 boys and 8 girls who were hospitalized and met the diagnostic criteria of the American Academy of Pediatrics for bronchiolitis^20^. Inclusion criteria for the bronchiolitis group: (1) younger than 2 years old with first episode of wheezing; (2) cough, shortness of breath; (3) crackles, use of accessory muscles, and/or nasal flaring; (4) excluding congenital heart disease, foreign matter in the airways, tuberculosis, and other congenital airway malformations that can cause asthma.

The control group consisted of 12 boys and 8 girls during the same period. Inclusion criteria for the normal control group: (1) no fever and infectious diseases within 4 weeks before the physical examination in our hospital; (2) no previous history of wheezing, allergies (personal and family allergies) and history of asthma; (3) no autoimmune diseases, immune deficiency, tumors and heart disease; (4) not use immune-suppressants and hormones.

### Sample collection and preservation

Peripheral venous blood was collected when infants with bronchiolitis were admitted to hospital. 2 ml was taken to a conventional procoagulant tube, and the serum was separated and stored in a refrigerator at − 20 °C. For these samples, we used Elisa and Liquidchip technology to test the expression levels of IL-9, IL-5, IL-13, IL-33, IgE, and TSLP. 2 ml was taken to EDTA anticoagulant tube, separated mononuclear cells and stored in − 80 °C ultra-low temperature refrigerator. For these samples, we used real-time quantitative RT-PCR to detect the mRNA expression levels of T1/ST2, IL-17RB, TSLP, RORα, and GATA-3. For healthy children in the control group, the method is the same as above.

### Experimental procedure

Before conducting the experiment, take the specimen from the − 20 °C refrigerator and − 80 °C refrigerator, and equilibrate to room temperature. Take the kit from the refrigerator and equilibrate at room temperature for at least 30 min.

### Detection of IgE

1 ml serum was sent to the laboratory of Xiangya Hospital for testing.

### Elisa

Human TSLP Elisa (R&D, USA) was performed according to the manufacturer’s protocol and analyzed using a microplate reader (R&D, USA).

### Liquidchip technology

Liquidchip technology was performed as described^21^ to detect the expression of IL-5, IL-9, IL-13, and IL-33. Firstly, thawed samples thoroughly, then mixed and centrifuged at 3000 *g* for 5 min. Lay out the plate according to protocols, then incubated overnight at 4 °C with shaking gently. The plate was blocked for 1 h with second antibody at room temperature, added 25ul Streptavidin-Phycoerythrin to each well and incubated for 30 min. Finally placed the plate into Luminex 200 (Luminex, USA), saved the results and calculated the cytokine concentration in the samples.

### Real-time PCR assay

Total RNA was isolated from peripheral blood mononuclear cells with trizol reagent. 0.5 μg total RNA was reverse transcribed with Transcriptor First Strand cDNA Synthesis Kit (Takara, USA) based on manufacturer's instructions. The real-time PCR was performed using SYBR Premix DimerEraser (Takara, USA) according to the manufacturer’s instructions. The reaction was carried out in a Roche LightCycler 480 Sequence Detection System (Roche, Germany). The results were calculated using the 2-delta delta Ct method, allowing for the normalization to Glyceraldehyde-3-phosphate dehydrogenase (GAPDH) with the calibrator set to a value of 1. The pairs of primer are listed as follows: T1/ST2: 5′-TTGCTTCACCCAGCAATTAAA-3′ (forward) and 5′-TTCAAACCAAAGGAACATGACC-3′ (reverse); IL17RB: 5′-GTCCCAGTGAGAACTCTCAA-3′ (forward) and 5′- CTAAAGTAGACCACCACGTA-3′(reverse); TSLP: 5′-AGAGCACTTA CTGTGGAAC-3′ (forward) and 5′- AAGAAACTGCCTATTCAGCTA-3′ (reverse); GATA3: 5′- GCTGTAAGGCATGAAGGAT-3′ (forward) and 5′- CTGGCAGTTTGTCCATTTGA-3′ (reverse); RORα: 5′- AGTAAGCCA AGCCTTACG-3′ (forward) and 5′- TCCTCAAGACCTACACACAAT-3′ (reverse) GAPDH: 5′- TGTTGCCATCAATGACCCCTT-3′(forward) and 5′- CTCCACGACGTACTCAGCG -3′ (reverse).

### Statistical analysis

Data were analyzed with SPSS 26.0 statistical software. Normal distribution was tested by student’s t test and the results were expressed as mean ± standard deviation ($\stackrel{-}{\mathrm{x}}$ ± s); the skewed distribution was tested by ranked sum test and the results are expressed in M (QR); *p* < 0.05 was considered significant.

## Results

### TSLP, T1/ST2, IL-17RB, RORα, and GATA-3 mRNA expression levels in peripheral blood of the bronchiolitis group (n = 26) and the control group (n = 20)

**Figure 1:**
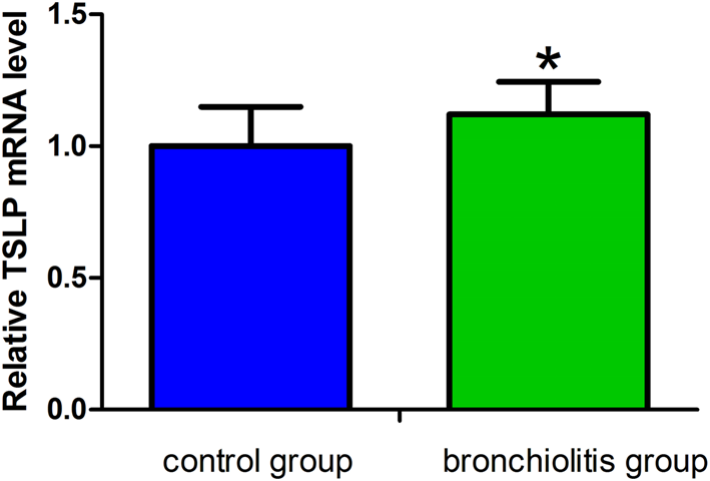
Comparison of TSLP mRNA expression levels in peripheral blood between control group and bronchiolitis group. The mRNA expression levels of TSLP in the bronchiolitis group were greater than control group (*P < 0.05).

**Figure 2:**
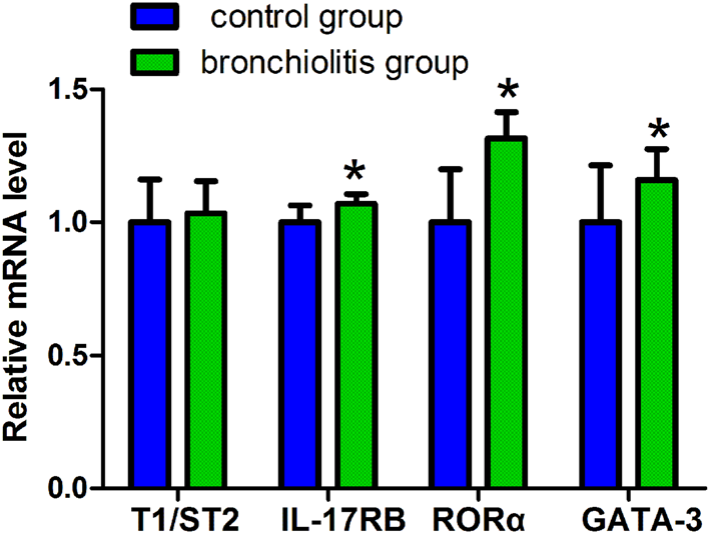
The mRNA expression levels of T1/ST2, IL-17RB, RORα, GATA-3 in control group and bronchiolitis group. IL-17RB, RORα, and GATA-3 mRNA expression levels in the bronchiolitis group were greater than control group (*P < 0.05), while there was no difference in T1/ST2 mRNA expression level between two groups (*P* > 0.05).

The mRNA expression levels of TSLP in the peripheral blood of the bronchiolitis group were greater than control group (16.98 ± 2.12 vs 15.07 ± 2.25, *P* < 0.05, see Fig. 1). At the same time, IL-17RB (9.83 ± 0.35), RORα (7.18 ± 0.71), and GATA-3 (4.86 ± 0.66) mRNA expression levels in the bronchiolitis group were greater than those (9.19 ± 0.58, 5.46 ± 1.09, and 4.19 ± 0.90, respectively) in the control group, and the differences between the two groups were statistically significant (*P* < 0.05, see Fig. 2).

There was no difference in T1/ST2 (IL-33R) mRNA expression level between the bronchiolitis group and the control group, and there was no statistical significance (5.59 ± 0.68 vs 5.41 ± 0.87, *P* > 0.05, see Fig. 2).

### Protein expression of IgE, IL-5, IL-9, IL-13, IL-33, and TSLP in peripheral blood of the bronchiolitis group (n = 26) and the control group (n = 20)

**Figure 3:**
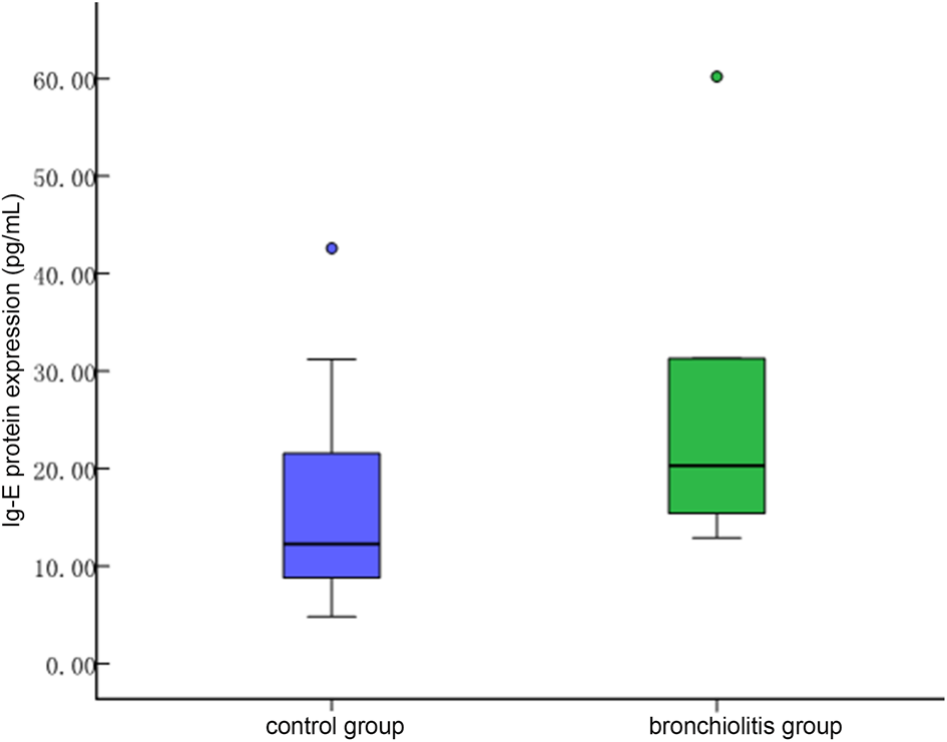
Ig-E protein levels of peripheral blood in control group and bronchiolitis group. There was no significance between two groups (*P* > 0.05).

IgE was not statistically significant between the bronchiolitis group and the control group [19.05 (14.15) vs 14.85 (20.2), *P* > 0.05, see Fig. 3].

**Figure 4:**
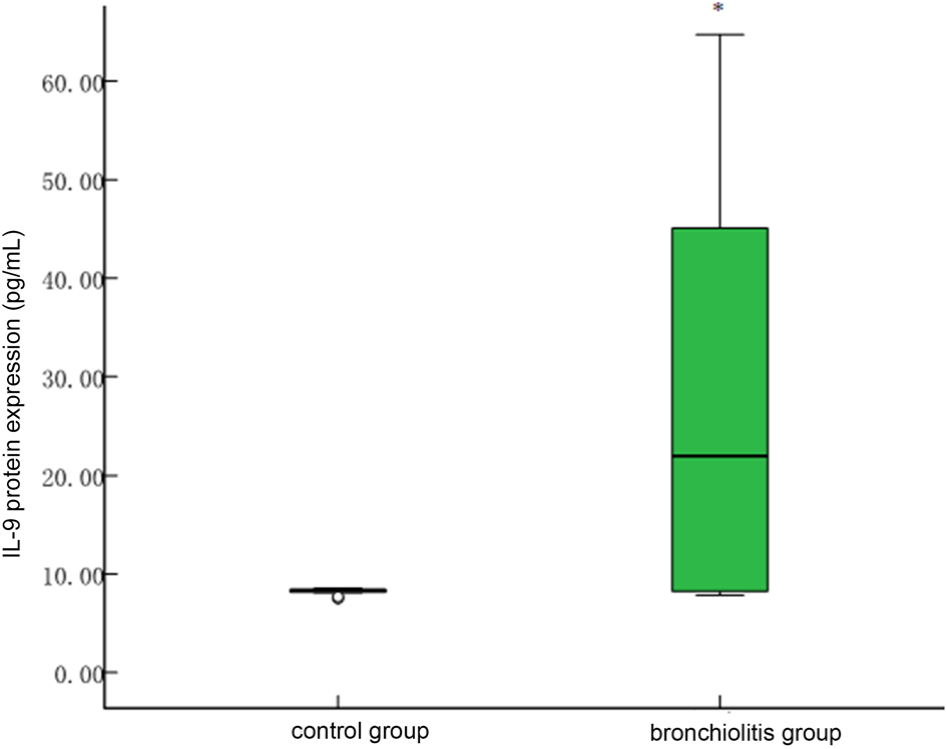
Comparison of IL-9 protein levels in peripheral blood between control group and bronchiolitis group. The protein expression level of IL-9 in bronchiolitis group was higher than control group (*P < 0.05).

**Figure 5:**
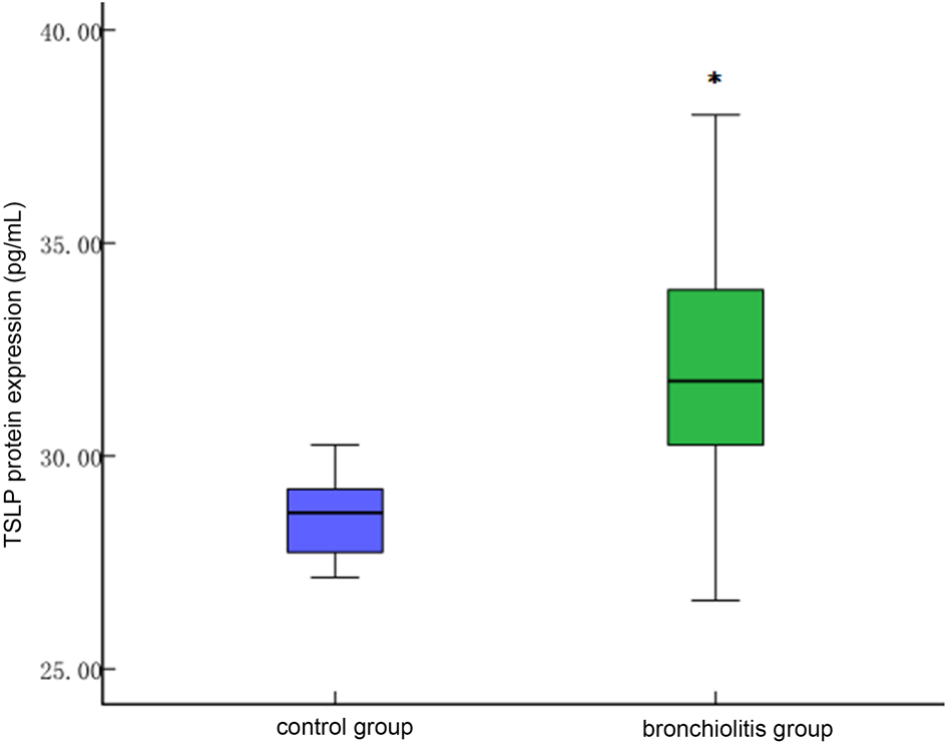
TSLP protein levels in peripheral blood between control group and bronchiolitis group. The protein expression level of TSLP in bronchiolitis group was higher than control group, which is in consistent with mRNA level (*P < 0.05).

**Figure 6:**
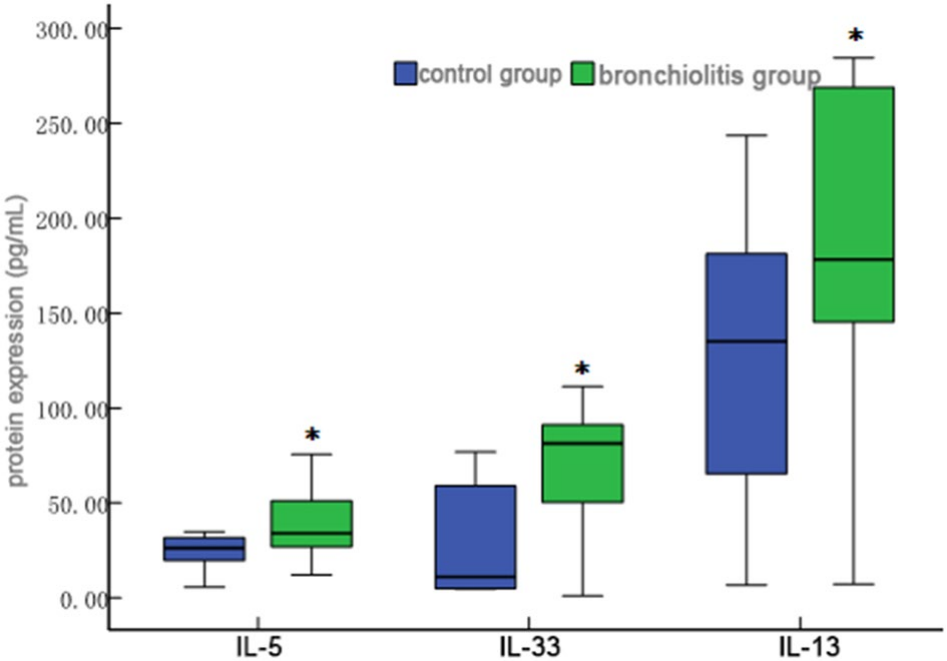
IL-5, IL-33 and IL-13 protein levels in control group and bronchiolitis group. IL-5, IL-33 and IL-13 in peripheral blood of the bronchiolitis group were higher than those in the control group (*P < 0.05).

The protein expression levels of IL-5 [41.99 (21.11)], IL-9 [27.04 (37.51)], IL-13 [184.05 (132.81)], IL-33 [83.70 (46.69)], and TSLP [31.42 (5.41)] in peripheral blood of the bronchiolitis group were higher than those [25.70 (19.64), 8.30 (0.54), 121.75 (176.13), 11.23 (55.31), and 28.76 (2.56) respectively] in the control group, and the differences between the two groups were statistically significant (*P* < 0.05, see Figs. 4, 5 and 6).

## Discussion

Researchers have found that in asthma patients, ILC2 differentiates and matures through specific transcription factors RORα and GATA-3, and under the stimulation of IL-25, IL-33 and TSLP, it secretes a large amount of type 2 cytokines IL-5, IL-9, IL-13^22^, which is immunological mechanism of pathogenicity. ROR is a key transcription factor for ILC development and maturity, which is divided into RORα and RORγt. RORα is a specific transcription factor for ILC2 development^17–19^. Similarly, GATA-3 plays an important role in the differentiation and maturation of ILC2 cells, which can be produced by ILC2 and common lymphoid precursor (CLP). It has been confirmed that the level of GATA-3 produced by CLP is extremely low, and the high level of GATA-3 is expressed by ILC2 cells, which is regarded as a specific marker^23^. Klein and his colleagues have found that in GATA-3 deficient chimeric mice, when IL-25 and IL-33 are used for intervention therapy, their ILCs have no increased expression and cannot secrete type 2 cytokines^24^. Our study measured the levels of RORα and GATA-3 in the peripheral blood of patients with bronchiolitis. The results showed that the expression of transcription factors RORα and GATA-3 in the bronchiolitis group was higher than that in the normal control group (*P* < 0.05). Based on RORα and GATA-3 as the specific markers of ILC2, it is suggested that ILC2s increase and activate in the peripheral blood of patients with bronchiolitis.

TSLP plays an important role in the primary recognition of pathogenic microorganisms and the occurrence of type 2 adaptive immune response. Besides, TSLP synergy with IL-33 and IL-25 is the key for type 2 innate immune response^25^. TSLP regulates the differentiation of CD4^+^ naive T lymphocytes to Th2 cells by inducing dendritic cells to produce IL-4, IL-5, IL-13, etc. Animal experiments have found that the specific expression of TSLP can induce AHR, airway inflammation, goblet cell hyperplasia, and lack of TSLP cannot produce allergen-induced lung inflammatory response^26,27^. It is suggested that TSLP is involved in airway inflammatory diseases, which is in accordance with Liu’s research^28^. Although TSLP acts alone on ILC2s to produce low levels of IL-5 and IL-13, synergy with IL-33 and IL-25 can promote the production of type 2 cytokines such as IL-5 and IL-13 and participate in airway inflammation and AHR.

IL-25 can specifically bind to IL-17RB (IL-25R) on ILC2s, prompting ILC2s to secrete large amounts of type 2 cytokines (IL-4, IL-5, IL-9 and IL-13)^29^. IL-25 can also amplify Th2 cell-mediated type 2 immune response^30^. Application of IL-25 monoclonal antibody in animal experiment can reduce the AHR and airway inflammation and inhibit airway remodeling^31^.

T1/ST2 is a specific receptor for IL-33, which is expressed on the surface of ILC2s cells and Th2 cells. Studies on rats have found that their ILC2 cells have IL-33 receptors, which can bind to IL-33, respond quickly, and produce IL-13 and IL-5^32^. Administration of IL-33 to experimental mice lacking T cells can cause airway eosinophilic inflammation^33^. IL-33 acts on ILC2 cells and stimulates it to secrete a large amount of type 2 cytokines^22^, leading to the occurrence of asthma.

Our study found that compared with the control group, the levels of IL-17RB, TSLP, and IL-33 in the bronchiolitis group were significantly increased. Although they can also participate in the pathogenesis of asthma through the Th2 cells pathway, which is not specific to ILC2 cells, this study found that the ILC2 specific transcription factors RORα and GATA-3 in the bronchiolitis group were significantly higher than the control group, indicating that ILC2 has been polarized. Therefore, IL-17RB, TSLP, IL-33 may participate in the development of bronchiolitis through ILC2s cells.

In the co-immunoprecipitation experiment, IL-33 binds to T1/ST2, activates downstream signaling molecules, and promotes the secretion of type 2 cytokines. Beltran et al.^34^ found that IL-33 in the nucleus can inhibit transcription factors and reduce the expression of pro-inflammatory factors, indicating that IL-33 has a dual role, on the one hand, it promotes the secretion of inflammatory factors, on the other hand, it inhibits transcription. For T1/ST2 there was no difference between the bronchiolitis group and the normal control group (*P* > 0.05), while IL-33 was significant different between the two groups (*P* < 0.05). It should be considered whether IL-33 has transcriptional repression in asthma and bronchiolitis. It has not been reported so far and needs further exploration for related mechanism.

Previous studies have suggested that IL-5, IL-9, and IL-13 are only Th2-derived cytokines, but in recent years, a large number of studies have confirmed that ILC2 can also secrete these cytokines^35,36^. IL-9 is a multi-effect cytokine, which is extremely important in the pathogenesis of asthma^37^. In this study, there was significant difference for IL-9 between the bronchiolitis group and the normal control group (*P* < 0.05). Mcnamava and Scruple reported that IL-9 increased in the peripheral serum of children with bronchiolitis, suggesting that IL-9 is involved in the airway inflammatory response of bronchiolitis^38,39^, which is consistent with our study.

In vivo, it was found that ILC2 is the main source of IL-13 in lung inflammation, and this response does not depend on T cells^40^. IL-13 can activate eosinophils, promote IgE secretion, participate in the maintenance of asthma inflammation, and induce small airway remodeling and AHR, which plays an important role in the development of asthma. The expression level of IL-13 in this study was higher than that of the normal control group, suggesting that IL-13 may be involved in airway inflammation and AHR in children with bronchiolitis in the acute phase. Experimental study suggests that in allergic or non-allergic asthma, IL-13 will be a very promising target for asthma treatment^41^.

IL-5 participates in chronic airway inflammation by promoting eosinophil production, activation, chemotaxis and prolonging cell life, so it is called eosinophil differentiation factor^42^. In animal experiment, using mice induced by inhalation of ovalbumin as a model, then introducing anti-IL-5 antibodies, results revealed that lung eosinophils were significantly reduced. The study also found that in Balb/c mice knocked out of the eosinophil chemokine gene and IL-5 gene, eosinophil production is blocked and AHR cannot be formed^43^. In this study, the serum IL-5 in the bronchiolitis group and the normal control group were significant different (*P* < 0.05), indicating that children with bronchiolitis have eosinophil activation and AHR, which is in line with the Nenna et al. study^44^.

In this study, none of the children in the bronchiolitis group had personal or family history of allergies, so IgE had no difference in serum protein levels between two groups (*P* > 0.05), and the expression of IgE gradually decreased as the disease relapsed.

It should be noted that limitations are existing in this study. Firstly, the sample size is relatively small, which may lead to low statistic power for data analysis. Secondly, respiratory specimens were not collected, only peripheral blood and the etiological agent of bronchiolitis was not determined in all patients, which may affect the consistency of the research results. It would be interesting to compare cytokines levels and the levels of study genes expression between the RSV-infected vs the other etiological agents of bronchiolitis.

In summary, our study found that the specific transcription factors GATA-3, RORα and IL-17RB specifically expressed by the ILC2s in the bronchiolitis group had higher mRNA expression levels than the normal control group. The bronchiolitis group serum IL-5, IL-9, IL-13, IL-33, TSLP protein levels were higher than the normal control group. These all indicate that ILC2 is involved in the immunological pathogenesis of bronchiolitis, which provides a new target for the treatment of bronchiolitis.
